# Dimethyl 2-[2-(2,4,6-tri­chloro­phen­yl)hydrazin-1-yl­idene]butane­dioate

**DOI:** 10.1107/S160053681303242X

**Published:** 2013-12-04

**Authors:** M. K. Usha, Shobhitha Shetty, B. Kalluraya, Rajni Kant, Vivek K. Gupta, D. Revannasiddaiah

**Affiliations:** a Department of Studies in Physics, University of Mysore, Manasagangotri, Mysore 570 006, India; b Department of Studies in Chemistry, Mangalore University, Mangalagangotri, Mangalore 574 199, India; c Post-Graduate Department of Physics & Electronics, University of Jammu, Jammu Tawi 180 006, India

## Abstract

In the title compound, C_12_H_11_Cl_3_N_2_O_4_, the dihedral angle between the aromatic ring and the hydrazine (NH—N=C) grouping is 52.2 (3)°. The butanedioate groups exhibit planar conformations. An intra­molecular N—H⋯O hydrogen bond links the N—H group of the hydrazine to one of the meth­oxy groups of the butane­dioate moiety. In the crystal, mol­ecules are linked by C—H⋯O hydrogen bonds and π–π inter­actions are also observed [centroid–centroid separation = 3.535 (1) Å].

## Related literature   

For the pharmacological activity of halo-substituted derivatives, see: Kees *et al.* (1996 ▶). For the use of the title compound in the synthesis of pyrazoles, see: Palacios *et al.* (1999 ▶). For the biological activity of pyrazoles, see: Palacios *et al.* (1999 ▶); Lee *et al.* (2003 ▶); Nithinchandra *et al.* (2012 ▶); Genin *et al.* (2000 ▶); Reddy *et al.* (2008 ▶); Kees *et al.* (1996 ▶). For a related structure, see: Huang *et al.* (2011 ▶).
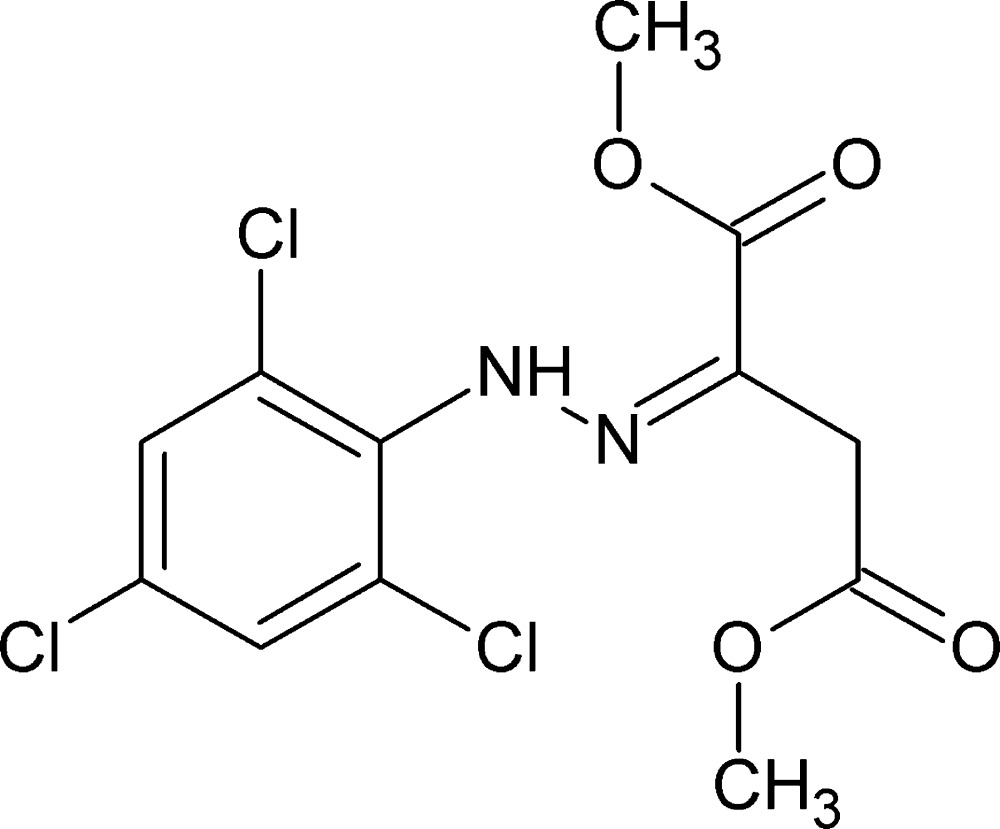


## Experimental   

### 

#### Crystal data  

C_12_H_11_Cl_3_N_2_O_4_
*M* *_r_* = 353.58
Orthorhombic, 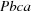

*a* = 7.0182 (5) Å
*b* = 16.0165 (12) Å
*c* = 26.7488 (15) Å
*V* = 3006.8 (4) Å^3^
*Z* = 8
Mo *K*α radiation
μ = 0.63 mm^−1^
*T* = 293 K
0.30 × 0.20 × 0.20 mm

#### Data collection  

Oxford Diffraction Xcalibur Sapphire3 diffractometer
Absorption correction: multi-scan (*CrysAlis RED*; Oxford Diffraction, 2010 ▶) *T*_min_ = 0.716, *T*_max_ = 1.000
7083 measured reflections
2954 independent reflections
1844 reflections with *I* > 2σ(*I*)
*R*_int_ = 0.040

#### Refinement  

*R*[*F* ^2^ > 2σ(*F* ^2^)] = 0.048
*wR*(*F* ^2^) = 0.118
*S* = 1.03
2954 reflections
195 parameters
H atoms treated by a mixture of independent and constrained refinement
Δρ_max_ = 0.26 e Å^−3^
Δρ_min_ = −0.24 e Å^−3^

### 

Data collection: *CrysAlis PRO* (Oxford Diffraction, 2010 ▶); cell refinement: *CrysAlis PRO*; data reduction: *CrysAlis PRO*; program(s) used to solve structure: *SHELXS97* (Sheldrick, 2008 ▶); program(s) used to refine structure: *SHELXL97* (Sheldrick, 2008 ▶); molecular graphics: *ORTEP-3 for Windows* (Farrugia, 2012 ▶); software used to prepare material for publication: *PLATON* (Spek, 2009 ▶).

## Supplementary Material

Crystal structure: contains datablock(s) I, New_Global_Publ_Block. DOI: 10.1107/S160053681303242X/sj5364sup1.cif

Structure factors: contains datablock(s) I. DOI: 10.1107/S160053681303242X/sj5364Isup2.hkl

Supporting information file. DOI: 10.1107/S160053681303242X/sj5364Isup3.cml

Additional supporting information:  crystallographic information; 3D view; checkCIF report

## Figures and Tables

**Table 1:** Hydrogen-bond geometry (Å, °)

*D*—H⋯*A*	*D*—H	H⋯*A*	*D*⋯*A*	*D*—H⋯*A*
N10—H10⋯O15	0.87 (3)	2.38 (3)	3.047 (3)	133 (3)
C5—H5⋯O19^i^	0.93	2.51	3.397 (4)	159

Symmetry code: (i) 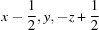.

**Fig. 1.**
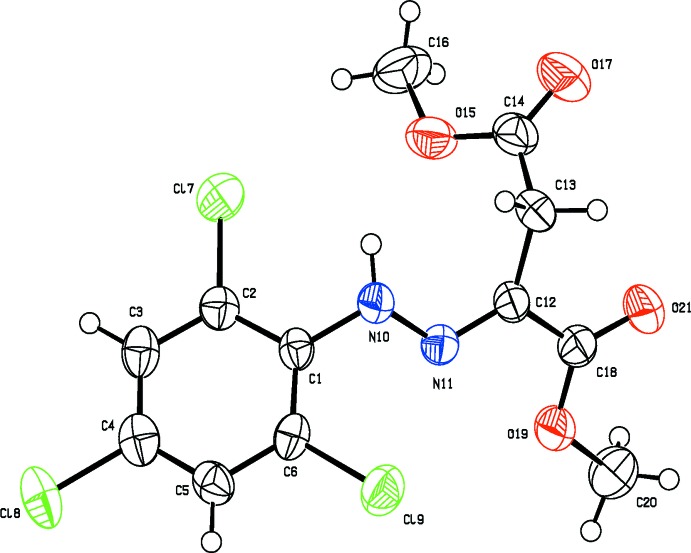
ORTEP view of the molecule with the atom-labeling scheme. The thermal ellipsoids are drawn at the 40% probability level. H atoms are shown as small spheres of arbitrary radius.

**Fig. 2.**
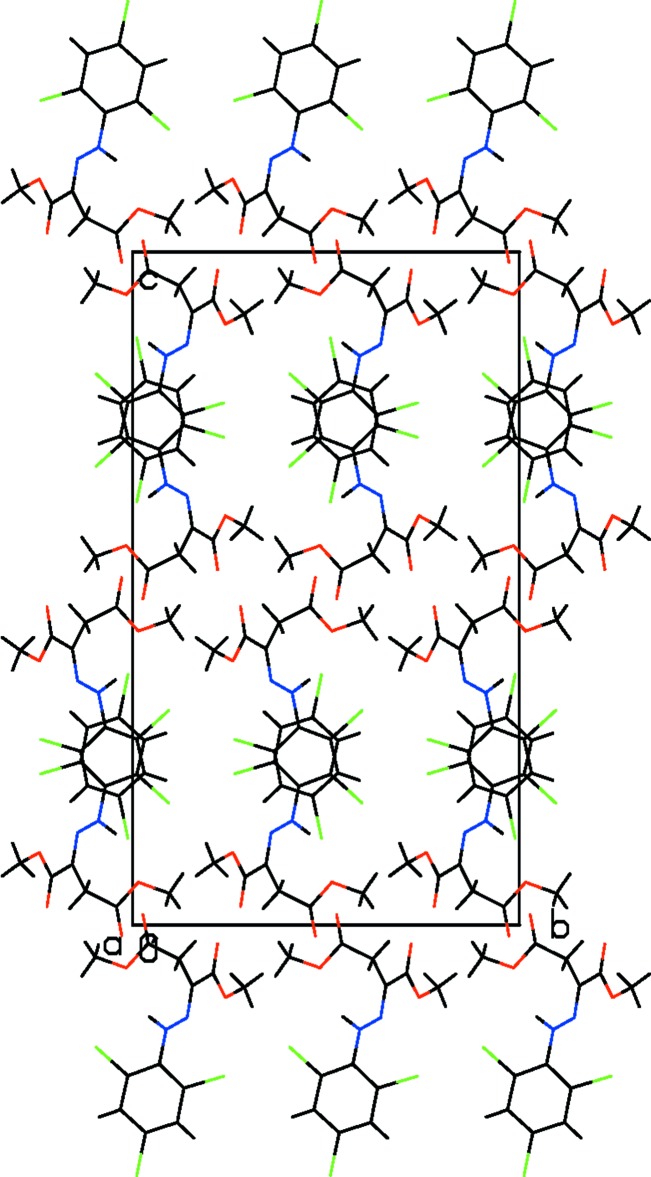
The packing arrangement of molecules viewed along the a axis.

## supplementary crystallographic information

### 1. Comment

It has been reported in the literature that halo substituted derivatives possess significant pharmacological activity (Kees *et al.*, 1996). Also the title compound can be used as an intermediate for the synthesis of pyrazoles (Palacios *et al.*, 1999). Aryl pyrazoles have antimicrobial (Palacios *et al.*, 1999, Lee *et al.*, 2003), anti-inflammatory (Nithinchandra *et al.*, 2012) and non-nucleoside HIV-I reverse transcriptase inhibitor activity (Genin *et al.*, 2000). Furthermore, pyrazoles with a wide array of substituted groups were reported to be selective inhibitors of cyclooxygenase (Reddy *et al.*, 2008) and also exhibit antidiabetic properties (Kees *et al.*, 1996).

In the title compound, C_12_H_11_C_13_N_2_O_4_, the trichlorophenyl ring is planar (r.m.s. deviation 0.018 Å); the largest deviation from the mean plane is 0.02 (3) Å for atom C1. The bond distances in the title compound are comparable to those observed in the closely related structure (E)-benzaldehyde (2,4,6-trichlorophenyl) hydrazone (Huang *et al.*, 2011). The C1—N10—N11—C12 torsion angle of the atoms joining the trichlorophenyl ring and the butanedioate group is 175.9 (3). An intramolecular N10–H10···O15 hydrogen bond links the N–H group of the hydrazine to one of the methoxy groups of the butanedioate moiety. In the crystal, molecules are stacked along the *a* axis by C5—H5..O19 hydrogen bonds and π–π interactions between adjacent trichlorophenyl rings [centroid–centroid separation = 3.535 (1) Å, interplanar spacing = 3.494 Å, centroid shift = 0.53 Å, symmetry code: -1/2 + *x*,*y*,1/2 - *z*].

### 2. Experimental

The title compound was prepared by refluxing a mixture of trichlorophenyl hydrazine (0.01 mol) and dimethylacetylene dicarboxylate (0.01 mol) in a 10 ml toluene solution for 4 h. The completion of the reaction was monitored by thin layer chromatography. After completion the solvent was evaporated under reduced pressure and the white solid obtained was recrystallized from ethanol.

### 3. Refinement

Atom H10 attached to N10 was located in a difference map and refined isotropically. The remaining H atoms were positioned geometrically and were refined as riding on their parent C atoms, with C—H distances of 0.93–0.97 Å; and with *U*_iso_(H) = 1.2*U*_eq_(C), except for the methyl groups where *U*_iso_(H) = 1.5*U*_eq_(C).

### Crystal data

**Table:**

C_12_H_11_Cl_3_N_2_O_4_	*F*(000) = 1440
*M**_r_* = 353.58	*D*_x_ = 1.562 Mg m^−^^3^
Orthorhombic, *P**b**c**a*	Mo *K*α radiation, λ = 0.71073 Å
Hall symbol: -P 2ac 2ab	Cell parameters from 1944 reflections
*a* = 7.0182 (5) Å	θ = 3.9–27.4°
*b* = 16.0165 (12) Å	µ = 0.63 mm^−^^1^
*c* = 26.7488 (15) Å	*T* = 293 K
*V* = 3006.8 (4) Å^3^	Block, white
*Z* = 8	0.30 × 0.20 × 0.20 mm

### Data collection

**Table:**

Oxford Diffraction Xcalibur Sapphire3 diffractometer	2954 independent reflections
Radiation source: fine-focus sealed tube	1844 reflections with *I* > 2σ(*I*)
Graphite monochromator	*R*_int_ = 0.040
Detector resolution: 16.1049 pixels mm^-1^	θ_max_ = 26.0°, θ_min_ = 3.9°
ω scans	*h* = −7→8
Absorption correction: multi-scan (*CrysAlis RED*; Oxford Diffraction, 2010)	*k* = −19→11
*T*_min_ = 0.716, *T*_max_ = 1.000	*l* = −32→30
7083 measured reflections	

### Refinement

**Table:**

Refinement on *F*^2^	Secondary atom site location: difference Fourier map
Least-squares matrix: full	Hydrogen site location: inferred from neighbouring sites
*R*[*F*^2^ > 2σ(*F*^2^)] = 0.048	H atoms treated by a mixture of independent and constrained refinement
*wR*(*F*^2^) = 0.118	*w* = 1/[σ^2^(*F*_o_^2^) + (0.0358*P*)^2^] where *P* = (*F*_o_^2^ + 2*F*_c_^2^)/3
*S* = 1.03	(Δ/σ)_max_ = 0.006
2954 reflections	Δρ_max_ = 0.26 e Å^−^^3^
195 parameters	Δρ_min_ = −0.24 e Å^−^^3^
0 restraints	Extinction correction: *SHELXL97* (Sheldrick, 2008), Fc^*^=kFc[1+0.001xFc^2^λ^3^/sin(2θ)]^-1/4^
Primary atom site location: structure-invariant direct methods	Extinction coefficient: 0.0029 (4)

### Special details

**Table:**

Experimental. *CrysAlis PRO*, Agilent Technologies, Version 1.171.36.28 (release 01–02-2013 CrysAlis171. NET) (compiled Feb 1 2013,16:14:44) Empirical absorption correction using spherical harmonics, implemented in SCALE3 ABSPACK scaling algorithm.
Geometry. All e.s.d.'s (except the e.s.d. in the dihedral angle between two l.s. planes) are estimated using the full covariance matrix. The cell e.s.d.'s are taken into account individually in the estimation of e.s.d.'s in distances, angles and torsion angles; correlations between e.s.d.'s in cell parameters are only used when they are defined by crystal symmetry. An approximate (isotropic) treatment of cell e.s.d.'s is used for estimating e.s.d.'s involving l.s. planes.
Refinement. Refinement of *F*^2^ against ALL reflections. The weighted *R*-factor *wR* and goodness of fit *S* are based on *F*^2^, conventional *R*-factors *R* are based on *F*, with *F* set to zero for negative *F*^2^. The threshold expression of *F*^2^ > σ(*F*^2^) is used only for calculating *R*-factors(gt) *etc*. and is not relevant to the choice of reflections for refinement. *R*-factors based on *F*^2^ are statistically about twice as large as those based on *F*, and *R*- factors based on ALL data will be even larger.

### Fractional atomic coordinates and isotropic or equivalent isotropic displacement parameters (Å^2^)

**Table:**

	*x*	*y*	*z*	*U*_iso_*/*U*_eq_	
Cl7	0.13759 (16)	0.09353 (6)	0.31855 (3)	0.0641 (3)	
Cl9	0.09570 (15)	−0.23591 (5)	0.27554 (3)	0.0589 (3)	
Cl8	0.16340 (14)	−0.01155 (7)	0.12827 (3)	0.0640 (3)	
O21	0.4517 (4)	−0.22006 (15)	0.47244 (7)	0.0604 (7)	
N11	0.2602 (4)	−0.14140 (16)	0.36258 (8)	0.0385 (6)	
N10	0.1266 (4)	−0.08714 (18)	0.34507 (9)	0.0406 (7)	
O19	0.5314 (3)	−0.24056 (15)	0.39230 (8)	0.0572 (7)	
O15	0.2372 (4)	0.01540 (15)	0.43653 (8)	0.0582 (7)	
C3	0.1464 (4)	0.0307 (2)	0.22574 (11)	0.0443 (8)	
H3	0.1510	0.0859	0.2150	0.053*	
C12	0.2654 (4)	−0.15507 (19)	0.41006 (10)	0.0385 (8)	
C1	0.1339 (4)	−0.0696 (2)	0.29355 (10)	0.0344 (7)	
O17	0.2154 (4)	−0.02745 (18)	0.51588 (9)	0.0796 (9)	
C6	0.1257 (4)	−0.13302 (19)	0.25794 (11)	0.0385 (8)	
C5	0.1340 (4)	−0.1159 (2)	0.20686 (11)	0.0429 (8)	
H5	0.1315	−0.1589	0.1835	0.051*	
C13	0.1318 (5)	−0.1207 (2)	0.44935 (11)	0.0459 (9)	
H13A	0.0081	−0.1111	0.4342	0.055*	
H13B	0.1158	−0.1622	0.4754	0.055*	
C4	0.1460 (4)	−0.0342 (2)	0.19178 (12)	0.0459 (9)	
C2	0.1399 (4)	0.0117 (2)	0.27610 (11)	0.0401 (8)	
C18	0.4236 (5)	−0.2085 (2)	0.42838 (12)	0.0437 (8)	
C14	0.1996 (5)	−0.0411 (2)	0.47251 (12)	0.0508 (9)	
C16	0.3100 (6)	0.0954 (2)	0.45282 (14)	0.0744 (12)	
H16A	0.3322	0.1303	0.4242	0.112*	
H16B	0.4275	0.0874	0.4706	0.112*	
H16C	0.2187	0.1217	0.4744	0.112*	
C20	0.6937 (6)	−0.2892 (3)	0.40768 (13)	0.0762 (13)	
H20A	0.7602	−0.3090	0.3787	0.114*	
H20B	0.6515	−0.3358	0.4273	0.114*	
H20C	0.7775	−0.2550	0.4273	0.114*	
H10	0.096 (5)	−0.044 (2)	0.3640 (11)	0.050 (10)*	

### Atomic displacement parameters (Å^2^)

**Table:**

	*U*^11^	*U*^22^	*U*^33^	*U*^12^	*U*^13^	*U*^23^
Cl7	0.0891 (8)	0.0417 (5)	0.0615 (6)	−0.0035 (5)	0.0025 (5)	−0.0015 (4)
Cl9	0.0786 (7)	0.0401 (5)	0.0580 (6)	−0.0024 (5)	−0.0102 (4)	0.0033 (4)
Cl8	0.0631 (6)	0.0898 (8)	0.0393 (5)	−0.0039 (6)	−0.0063 (4)	0.0186 (5)
O21	0.0808 (18)	0.0676 (18)	0.0327 (13)	0.0156 (15)	−0.0090 (12)	0.0032 (11)
N11	0.0441 (16)	0.0355 (15)	0.0359 (14)	0.0012 (13)	−0.0024 (11)	0.0004 (12)
N10	0.0456 (16)	0.0408 (16)	0.0353 (16)	0.0085 (14)	0.0012 (12)	0.0040 (14)
O19	0.0691 (16)	0.0619 (17)	0.0406 (13)	0.0275 (14)	0.0008 (12)	0.0060 (11)
O15	0.0717 (18)	0.0510 (16)	0.0519 (14)	−0.0054 (14)	0.0097 (13)	−0.0132 (12)
C3	0.0340 (17)	0.050 (2)	0.049 (2)	−0.0022 (17)	−0.0002 (14)	0.0149 (18)
C12	0.0480 (19)	0.0361 (18)	0.0314 (16)	−0.0027 (16)	−0.0009 (13)	0.0017 (13)
C1	0.0289 (16)	0.0398 (19)	0.0344 (17)	0.0020 (15)	0.0001 (12)	0.0065 (14)
O17	0.109 (2)	0.090 (2)	0.0399 (14)	0.018 (2)	−0.0080 (14)	−0.0129 (15)
C6	0.0330 (16)	0.0365 (19)	0.0461 (19)	0.0000 (15)	−0.0038 (14)	0.0096 (15)
C5	0.0390 (18)	0.055 (2)	0.0346 (18)	0.0054 (18)	−0.0061 (14)	−0.0010 (16)
C13	0.055 (2)	0.050 (2)	0.0328 (17)	0.0003 (18)	0.0049 (14)	−0.0002 (16)
C4	0.0306 (17)	0.060 (2)	0.0470 (19)	−0.0008 (18)	−0.0043 (14)	0.0105 (19)
C2	0.0366 (17)	0.0421 (19)	0.0415 (19)	−0.0009 (16)	0.0009 (14)	0.0031 (15)
C18	0.058 (2)	0.0343 (18)	0.0390 (19)	−0.0038 (17)	−0.0011 (16)	−0.0001 (15)
C14	0.049 (2)	0.062 (2)	0.041 (2)	0.014 (2)	0.0062 (16)	−0.0017 (18)
C16	0.091 (3)	0.045 (2)	0.087 (3)	0.000 (2)	0.001 (2)	−0.016 (2)
C20	0.081 (3)	0.081 (3)	0.067 (3)	0.043 (3)	−0.002 (2)	0.008 (2)

### Geometric parameters (Å, º)

**Table:**

Cl7—C2	1.735 (3)	C12—C13	1.512 (4)
Cl9—C6	1.727 (3)	C1—C2	1.383 (4)
Cl8—C4	1.741 (3)	C1—C6	1.394 (4)
O21—C18	1.209 (3)	O17—C14	1.186 (3)
N11—C12	1.289 (3)	C6—C5	1.395 (4)
N11—N10	1.362 (3)	C5—C4	1.372 (5)
N10—C1	1.407 (4)	C5—H5	0.9300
N10—H10	0.88 (3)	C13—C14	1.495 (5)
O19—C18	1.329 (4)	C13—H13A	0.9700
O19—C20	1.440 (4)	C13—H13B	0.9700
O15—C14	1.348 (4)	C16—H16A	0.9600
O15—C16	1.447 (4)	C16—H16B	0.9600
C3—C2	1.382 (4)	C16—H16C	0.9600
C3—C4	1.380 (5)	C20—H20A	0.9600
C3—H3	0.9300	C20—H20B	0.9600
C12—C18	1.485 (4)	C20—H20C	0.9600
			
C12—N11—N10	117.8 (3)	H13A—C13—H13B	107.7
N11—N10—C1	116.1 (2)	C5—C4—C3	121.7 (3)
N11—N10—H10	118 (2)	C5—C4—Cl8	119.3 (3)
C1—N10—H10	115 (2)	C3—C4—Cl8	119.0 (3)
C18—O19—C20	116.9 (3)	C3—C2—C1	122.5 (3)
C14—O15—C16	116.7 (3)	C3—C2—Cl7	118.1 (3)
C2—C3—C4	118.4 (3)	C1—C2—Cl7	119.3 (2)
C2—C3—H3	120.8	O21—C18—O19	123.7 (3)
C4—C3—H3	120.8	O21—C18—C12	122.2 (3)
N11—C12—C18	116.4 (3)	O19—C18—C12	114.1 (3)
N11—C12—C13	127.3 (3)	O17—C14—O15	123.8 (4)
C18—C12—C13	116.3 (3)	O17—C14—C13	126.4 (4)
C2—C1—C6	117.1 (3)	O15—C14—C13	109.8 (3)
C2—C1—N10	121.3 (3)	O15—C16—H16A	109.5
C6—C1—N10	121.5 (3)	O15—C16—H16B	109.5
C1—C6—C5	121.6 (3)	H16A—C16—H16B	109.5
C1—C6—Cl9	120.9 (2)	O15—C16—H16C	109.5
C5—C6—Cl9	117.4 (3)	H16A—C16—H16C	109.5
C4—C5—C6	118.5 (3)	H16B—C16—H16C	109.5
C4—C5—H5	120.7	O19—C20—H20A	109.5
C6—C5—H5	120.7	O19—C20—H20B	109.5
C14—C13—C12	113.6 (3)	H20A—C20—H20B	109.5
C14—C13—H13A	108.8	O19—C20—H20C	109.5
C12—C13—H13A	108.8	H20A—C20—H20C	109.5
C14—C13—H13B	108.8	H20B—C20—H20C	109.5
C12—C13—H13B	108.8		
			
C12—N11—N10—C1	175.9 (3)	C4—C3—C2—C1	0.3 (5)
N10—N11—C12—C18	−174.7 (3)	C4—C3—C2—Cl7	−179.3 (2)
N10—N11—C12—C13	3.4 (5)	C6—C1—C2—C3	−2.9 (5)
N11—N10—C1—C2	−127.5 (3)	N10—C1—C2—C3	−179.8 (3)
N11—N10—C1—C6	55.8 (4)	C6—C1—C2—Cl7	176.7 (2)
C2—C1—C6—C5	3.5 (4)	N10—C1—C2—Cl7	−0.2 (4)
N10—C1—C6—C5	−179.6 (3)	C20—O19—C18—O21	−2.2 (5)
C2—C1—C6—Cl9	−174.0 (2)	C20—O19—C18—C12	176.6 (3)
N10—C1—C6—Cl9	2.9 (4)	N11—C12—C18—O21	173.9 (3)
C1—C6—C5—C4	−1.6 (4)	C13—C12—C18—O21	−4.3 (5)
Cl9—C6—C5—C4	176.0 (2)	N11—C12—C18—O19	−4.9 (4)
N11—C12—C13—C14	−92.3 (4)	C13—C12—C18—O19	176.8 (3)
C18—C12—C13—C14	85.7 (4)	C16—O15—C14—O17	3.2 (5)
C6—C5—C4—C3	−1.2 (5)	C16—O15—C14—C13	−178.2 (3)
C6—C5—C4—Cl8	178.2 (2)	C12—C13—C14—O17	−127.7 (4)
C2—C3—C4—C5	1.8 (5)	C12—C13—C14—O15	53.8 (4)
C2—C3—C4—Cl8	−177.6 (2)		

### Hydrogen-bond geometry (Å, º)

**Table:**

*D*—H···*A*	*D*—H	H···*A*	*D*···*A*	*D*—H···*A*
N10—H10···O15	0.87 (3)	2.38 (3)	3.047 (3)	133 (3)
C5—H5···O19^i^	0.93	2.51	3.397 (4)	159

Symmetry code: (i) *x*−1/2, *y*, −*z*+1/2.
